# The ACSL4 Network Regulates Cell Death and Autophagy in Diseases

**DOI:** 10.3390/biology12060864

**Published:** 2023-06-15

**Authors:** Fangquan Chen, Rui Kang, Jiao Liu, Daolin Tang

**Affiliations:** 1 DAMP Laboratory, The Third Affiliated Hospital of Guangzhou Medical University, Guangzhou 510120, China; 2 Guangzhou Municipal and Guangdong Provincial Key Laboratory of Protein Modification and Degradation, State Key Laboratory of Respiratory Disease, Guangzhou Medical University, Guangzhou 511436, China; 3 Department of Surgery, UT Southwestern Medical Center, Dallas, TX 75390, USA

**Keywords:** ACSL4, autophagy, apoptosis, cancer, ferroptosis

## Abstract

**Simple Summary:**

ACSL4 is an enzyme involved in the intracellular conversion of long-chain fatty acids and coenzyme A into fatty-acid coenzymes. It plays a vital role in various biological processes, including maintaining cell membrane structure, energy metabolism, and lipid metabolism. Recent studies have shed light on the involvement of ACSL4 in cell death pathways, autophagy regulation, and the development of human diseases. These findings position ACSL4 as a potential therapeutic target. This review provides an overview of the fundamental structure and mechanisms regulating ACSL4 at both the gene and protein levels, emphasizing its diverse biological functions. Additionally, we discuss the role of ACSL4 in different regulated cell death modalities, including apoptosis and ferroptosis, as well as its involvement in autophagosome formation. Furthermore, we explore potential modulators targeting ACSL4 and emphasize the importance of further research to comprehensively understand the clinical impact of ACSL4 in regulating various pathological conditions. By addressing concerns regarding the systemic impact of therapeutic approaches targeting ACSL4, we aim to pave the way for the development of effective treatments for human diseases.

**Abstract:**

Lipid metabolism, cell death, and autophagy are interconnected processes in cells. Dysregulation of lipid metabolism can lead to cell death, such as via ferroptosis and apoptosis, while lipids also play a crucial role in the regulation of autophagosome formation. An increased autophagic response not only promotes cell survival but also causes cell death depending on the context, especially when selectively degrading antioxidant proteins or organelles that promote ferroptosis. ACSL4 is an enzyme that catalyzes the formation of long-chain acyl-CoA molecules, which are important intermediates in the biosynthesis of various types of lipids. ACSL4 is found in many tissues and is particularly abundant in the brain, liver, and adipose tissue. Dysregulation of ACSL4 is linked to a variety of diseases, including cancer, neurodegenerative disorders, cardiovascular disease, acute kidney injury, and metabolic disorders (such as obesity and non-alcoholic fatty liver disease). In this review, we introduce the structure, function, and regulation of ACSL4; discuss its role in apoptosis, ferroptosis, and autophagy; summarize its pathological function; and explore the potential implications of targeting ACSL4 in the treatment of various diseases.

## 1. Introduction

Cells are the basic units of living organisms, and their various biological functions and death are essential for life activities. The fate of cells is determined by genetic clues and the external environment. One crucial determinant is how cells respond to oxidative stress, as most organisms rely on oxygen as an electron acceptor for redox-based biological processes [1]. The redox state of the membrane is a crucial determinant of cell death, including both apoptosis and non-apoptotic cell death. Apoptosis is a type of programmed cell death that occurs naturally as part of the normal development and maintenance of multicellular organisms [2]. Intrinsic apoptosis is initiated by various stimuli, including mitochondrial dysfunction, which triggers a series of events leading to the activation of caspases (proteases that break down cellular components) and ultimately resulting in cell death [3]. Ferroptosis, a non-apoptotic cell death, is driven by membrane lipid peroxidation. Although its sensitivity is regulated by multiple cellular metabolic pathways, lipid metabolism is among the predominant metabolic changes during ferroptosis [4]. Lipid metabolism is a dynamic process involving synthesis, storage, utilization, and peroxidation. Functionally, lipid synthesis not only provides raw materials for various types of cellular structures and biofilms, but also prevents ferroptosis through the production of monounsaturated fatty acids (MUFAs). In contrast, lipid peroxidation mediated by enzymatic pathways and the enzyme-independent Fenton reaction is directly involved in ferroptosis, and the integration of polyunsaturated fatty acids (PUFAs) into phospholipids by acyl-CoA synthetase long-chain family member 4 (ACSL4) is a crucial event in ferroptosis [5,6]. Inhibition of lipid peroxidation by pharmacological or genetic means significantly abolishes ferroptosis, and these unique features and mechanisms distinguish ferroptosis from other known cell death modalities, such as alkaliptosis, apoptosis, and necroptosis [7,8].

**Figure 1:**
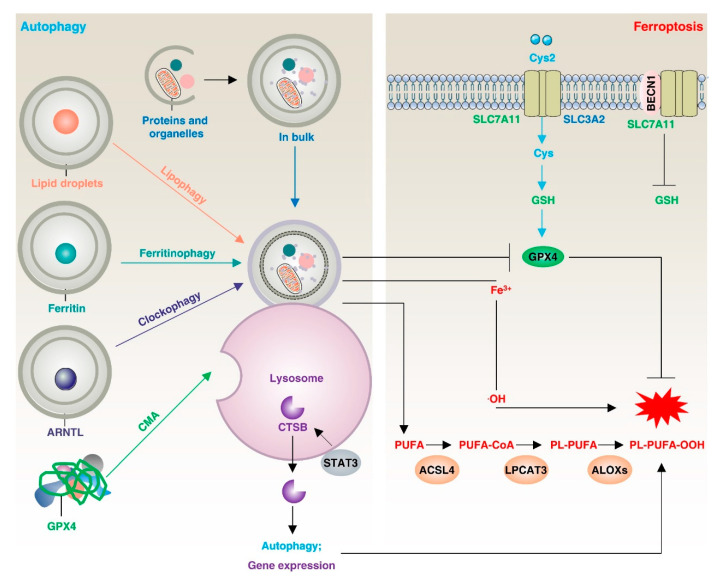
Autophagy-dependent ferroptosis. Macroautophagy/autophagy or selective autophagy (lipophagy, ferritinophagy, clockophagy, chaperone-mediated autophagy) promotes the degradation of organelles, ferritin, lipid droplets, or proteins (such as GPX4 and ARNTL) to increase intracellular Fe^2+^ or free fatty acids, promoting ferroptosis. In addition, BECN1 binding to SLC7A11 or cathepsin B release also promotes ferroptosis through lipid peroxidation. In this process, ACSL4 plays a crucial role by effectively binding long-chain polyunsaturated fatty acids (PUFAs) with coenzyme A, enabling their re-esterification into phospholipids through the action of lysophosphatidylcholine acyltransferase 3 (LPCAT3). This interaction further facilitates the promotion of ferroptosis.

Autophagy is an intrinsic degradation process in cells that plays a dual role in maintaining cellular homeostasis. In many cases, autophagy can remove unwanted proteins or organelles and recycle them to promote cell survival [9], while excessive or dysregulated autophagy can also induce cell death [10]. Accumulating evidence suggests that autophagic flux is increased during ferroptosis, and inhibition of autophagy rescues ferroptotic cell death. Established genetic and biochemical evidence suggests that the onset and execution of ferroptosis requires the autophagic machinery, including lipophagy, clockophagy, ferritinophagy, and beclin1 (BECN1)-dependent inhibition of solute carrier family 7 member 11 (SLC7A11), to trigger iron accumulation and/or lipid peroxidation [11,12] (Figure 1). More importantly, the induction of autophagy is dependent on lipid-derived biofilms, especially the endoplasmic reticulum (ER), which requires ACSL4 to perform functions such as lipid synthesis and activation [13].

This review provides an overview of the gene and protein structure of ACSL4, as well as the mechanisms involved in its regulation. We also focus on the content-dependent functions and roles of ACSL4 in different pathologies, as well as current ACSL4-targeted drugs. We believe that an in-depth understanding of ACSL4 is key to understanding the link between autophagy and cell death and will contribute to the subsequent precision treatment of related diseases.

## 2. Discovery of ACSL4

To date, five isoforms of human acyl-CoA synthase long-chain family members (ACSLs) have been identified (Table 1), including ACSL1, ACSL3, ACSL4, ACSL5, and ACSL6 [14]. The function of fatty-acid coenzyme A ligases has been understood since the 1960s [15]. In 1976, several studies showed a significant upregulation of fatty-acid ligase activity in the presence of fatty acids, suggesting that fatty-acid ligases mediate the lipid synthesis of fatty acids [16,17]. With the development of genetic technology, scientists began to uncover the diversity of fatty-acid coenzyme A ligases. In 1982, Wilson et al. discovered the possible existence of two long-chain acyl-CoA synthases in human platelets, one of which is specific for the prostaglandin precursors arachidonic acid and 8,11,14-eicosatrienoic acid [18]. In 1985, Nagamatsu et al. isolated and purified a long-chain acyl-CoA synthase from rat brain microsomes, and differences in substrate specificity between the liver enzyme and brain enzyme suggested that the long-chain acyl-CoA synthase contained several different isoforms [19]. In 1992, Abe et al. isolated a complementary DNA clone encoding the entire human long-chain acyl-CoA synthetase and determined that the enzyme encoded by this gene contains five domains [20]. In 1997, Kang et al. cloned a novel rat acyl-coenzyme A synthase preferentially utilizing arachidonic and eicosapentaenoic acids, named “ACS4”, which was detected in a wide range of tissues, with highest levels particularly in the adrenal gland [21]. At the same time, Cao et al. cloned the cDNA sequence of human *ACSL4* for the first time and used it to perform sequence analysis and functional characterization. They used expressed sequence tagging to identify a human brain cDNA containing the remainder of the coding region of *ACSL4* and predicted a 97% identity between the 670-amino-acid human and rat ACSL4 [22,23]. In 1998, Piccini et al. cloned the *ACSL4* cDNA and, using Northern blot analysis, found that ACSL4 is expressed as a 5 kb mRNA in a variety of tissues, although the transcript in the brain appears to be slightly larger [22]. In 2002, Meloni et al. found that ACSL4 was highly expressed in the human adult brain, especially in the cerebellum and hippocampus, and exhibited a distribution similar to that in mice [24]. Subsequent studies have shown that ACSL4 plays an important role in many biological processes, such as cell death, development, inflammation, autophagy, and metabolism [14,25,26,27,28].

In conclusion, the cloning of ACSL4 is the result of years of effort by multiple researchers and studies involving various fields, including gene sequencing, expression regulation, biological processes, and disease development. Further exploration of the biological functions and structures of ACSL4 will provide a critical basis for subsequent targeted therapies in clinical settings.

## 3. Structure of ACSL4 Gene and Protein

**Figure 2:**
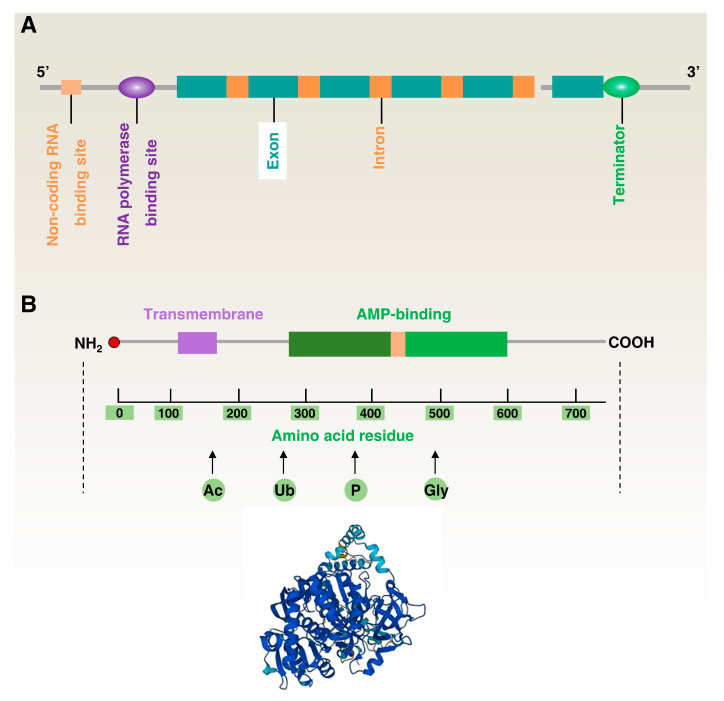
Structure of the ACSL4 gene and protein. (**A**) Simplified diagram of the ACSL4 gene. (**B**) Simplified diagram of the ACSL4 protein (top) and 3D model of ACSL4 protein.

ACSL4 is a protein-coding gene located on human chromosome Xq23.1, with a total length of approximately 90 kb and multiple exons and introns [29,30]. Its 5-prime flanking region lacks a typical TATA box but contains a CCAAT box and many transcription factor-binding sites, including peroxisome proliferator activated receptors (PPARs) [31], nuclear receptor subfamily 1 group H members (NR1Hs; also known as LXRs) [32], sp1 transcription factor (SP1) [33], transcription factor AP-2 (TFAP2) [34], nuclear factor kappa B subunit 1 (NFKB1; also known as NF-κB), and cAMP responsive element binding protein 1 (CREB1) [35,36], which regulate the transcriptional activity of the *ACSL4* gene and play important roles in fatty-acid metabolism. The *ACSL4* gene’s transcription start site is positioned on the X chromosome; specifically, at base 154,053,424 of the genomic sequence. This location is within the promoter region, which binds to the transcription factor and initiates transcription of the *ACSL4* gene. On the other hand, the transcription stop site is located at the end of the last exon [23,37]. Furthermore, the 5’ untranslated region (UTR) of the *ACSL4* gene contains several microRNA (miRNA) binding sites, and these miRNAs can regulate the expression level of ACSL4 by repressing the translation or degradation of *ACSL4* mRNA (Figure 2A) [38,39].

The human ACSL4 protein contains approximately 711 amino acid residues with a molecular weight of about 80 kDa and an isoelectric point of 8.66 [40]. Using crystallographic techniques, the ACSL4 protein structure can be divided into four regions: the N-terminal membrane-bound domain, the transmembrane structural domain, the adenylate-bound domain, and the C-terminal catalytic domain (Figure 2B) [20].

The N-terminal region is the most anterior part of ACSL4 and contains two unstable subregions (N1 and N2), which mainly consist of a segment containing several leucine amino acids. While the precise function of this region remains incompletely understood, it is speculated that its primary role is to facilitate the acylation reaction of fatty acids. This process involves the binding fatty acids to coenzyme A, resulting in the formation of acyl-coenzyme A. These compounds serve as a source of energy and building blocks for intracellular fatty-acid metabolism. Furthermore, it is possible that this region also plays a role in protein localization. The function of the transmembrane polypeptide is primarily to penetrate the membrane and connect the N-terminal and C-terminal structural domains, which contain a fatty-acid-binding pocket for long-chain fatty acids [41,42].

The adenosine monophosphate (AMP) adaptor region is the third structural domain of ACSL4, which is an adenosine-related structural domain used to link adenosine triphosphate (ATP) and CoA molecules. The structure of this region mainly consists of two substructures: the nucleotide-binding substructure and the CoA-binding substructure. These are linked together by a cantilever peptide to form the complete AMP junction region. The C-terminal structural domain is one of the most important structures of ACSL4 and is used to catalyze the acylation reaction, forming the ACSL4 catalytic active center. The structure of ACSL4 protein is very similar to other ACSLs, and its catalytic active center includes a conserved acylase kinetic triad (Lysine (Lys)-Aspartate (Asp)-Cysteine (Cys)). In this triad, Lys and Asp play protonation and deprotonation roles in catalyzing the acylation reaction, while Cys is the site of fatty-acid binding to CoA [43,44].

In the reaction catalyzed by long-chain fatty-acid CoA ligases, the ACSL4 protein provides a substrate for the β-oxidation of fatty acids by binding long-chain fatty acids to CoA to form acylation products. This domain contains many arginine residues that help to anchor coenzyme A and forms a basic pocket structure that allows it to accommodate fatty-acid molecules [45,46]. Furthermore, the ACSL4 protein contains several different modification sites, such as for phosphorylation, glycosylation, and ubiquitination, which are discussed later in this review.

Overall, the protein structure of ACSL4 is highly complex, with multiple structural domains forming a complete three-dimensional spatial structure. A detailed study of the ACSL4 protein structure is crucial for understanding its biological functions and regulatory mechanisms, which can help in the search for therapeutic approaches for related diseases and the development of related drugs. There are several splice variants of ACSL4 that may be involved in lipid metabolism disorders, adding to the complexity of this enzyme’s regulation and function. Further investigation into the various forms of ACSL4 may provide new insights into its roles in health and disease.

## 4. Function of ACSL4

**Figure 3:**
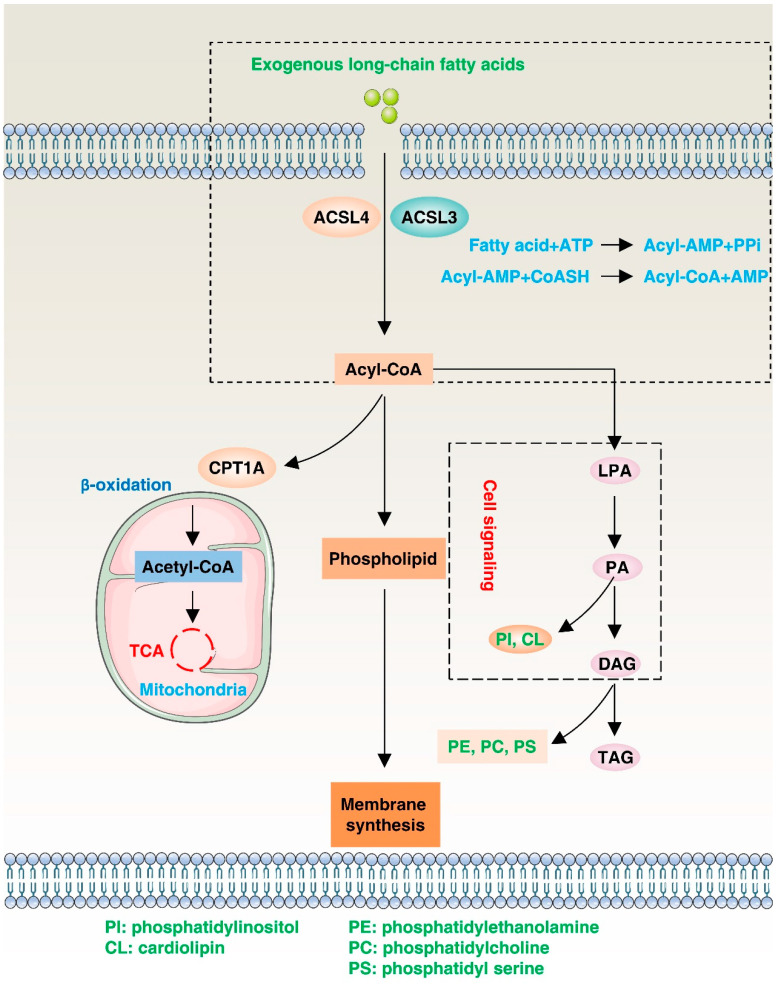
Function of ACSL4. ACSL4 catalyzes the conversion of exogenous long-chain fatty acids into acyl-coenzyme A. Acyl-CoA can then be transported into the mitochondria via carnitine palmitoyltransferase-1 (CPT1), where it can be converted to acetyl-CoA and participate in the tricarboxylic acid cycle to provide energy. Additionally, acyl-CoA can produce phosphatidylinositol, which contributes to membrane synthesis. Acyl-CoA can promote diacylglycerol acyltransferase (DGAT) and be involved in the synthesis of triacylglycerol (TAG) in lipid droplets (LDs) for lipid storage. Intermediates such as lyso-phosphatidic acid (LPA), phosphatidic acid (PA), and diacylglycerol (DAG) may initiate signaling cascades, and PA and DAG can also serve as precursors to all glycerophospholipids.

ACSL4’s location and function in the cell membrane make it a critical regulator of lipid metabolism, signal transduction, and other cellular processes (Figure 3). In Section 4, we will focus on ACSL4’s functions in terms of its role in the β-oxidation reaction, lipid synthesis pathway, and cellular signaling.

The β-oxidation reaction is a lipid metabolic pathway that progressively breaks down fatty acids into smaller acyl-CoA molecules to produce energy and metabolites [47]. In this process, the acyl-CoA molecule enters the mitochondria and is progressively cleaved into two-carbon acetyl-CoA molecules by a series of enzymes, producing energy molecules such as flavine adenine dinucleotide (FADH_2_) and nicotinamide adenine dinucleotide (NADH) [48]. These energy molecules further participate in the tricarboxylic acid cycle to produce more ATP energy. ACSL4’s ability to bind free fatty acids to CoA enables fatty acids to enter the mitochondria to participate in β-oxidation reactions [49]. The expression level of ACSL4 is closely related to the development of fatty liver and tumors. Therefore, targeting ACSL4 could be a potential strategy for treating metabolic diseases and cancer. In the future, it is expected that there will be efforts to characterize the interactions between ACSL4 and other enzymes involved in β-oxidation, as well as to examine the effects of ACSL4 mutations or dysregulation on β-oxidation efficiency and lipid metabolism in various diseases.

As a ligase, ACSL4 is responsible for linking long-chain fatty acids to CoA in cells to form the activated form of fatty acids, fatty acyl-coenzyme A (FA-CoA), which is a crucial component of the lipid synthesis pathway. Lipid synthesis is the process of converting carbohydrates and proteins into complex organic compounds such as lipids and phospholipids in living organisms [50,51]. In this pathway, ACSL4 can convert fatty acids into an activated form capable of participating in the synthesis of more complex lipids, such as phospholipids and other substances that provide energy and building blocks for cellular structures. ACSL4 also plays a role in other aspects of the lipid synthesis pathway. For instance, ACSL4-catalyzed FA-CoA is involved in the synthesis of triacylglycerol, which helps to store energy in the body, and the synthesis of saturated fatty acids, which is important for the physical properties of cell membranes and metabolic regulation [51]. Thus, ACSL4 plays a crucial role as a central regulator in the lipid synthesis pathway by facilitating the production of active substances required for lipid synthesis and participating in various complex biochemical reactions.

In addition to its roles in β-oxidation and lipid synthesis, ACSL4 also plays a role in cell signaling pathways affecting biological processes such as cell growth [52], differentiation [53], and death [54]. ACSL4’s involvement in these pathways mainly occurs through the regulation of synthesis and metabolism of lipid signaling molecules. Phosphatidylinositol and sphingomyelin are important lipid signaling molecules, and ACSL4 can promote their synthesis and metabolism to regulate cell signaling pathways [53]. Conversely, some key signaling factors can also regulate ACSL4, promoting cell survival. For example, reduced expression of sirtuin 3 (SIRT3) in patients with gallbladder cancer leads to the inhibition of ACSL4 through AKT serine/threonine kinase (AKT/PKB) signaling, in contrast to the normal tissues surrounding the cancer. This inhibition of ACSL4 suppresses ferroptosis while promoting epithelial–mesenchymal transition and invasive activity. Conversely, by blocking AKT activity in *SIRT3*-knockdown cells, ACSL4 expression is induced, thereby promoting ferroptosis [55]. Gaining a deeper understanding of ACSL4’s role in cell signaling transduction could lead to new insights and methods for preventing and treating diseases associated with cell death.

## 5. Modulation of ACSL4

**Figure 4:**
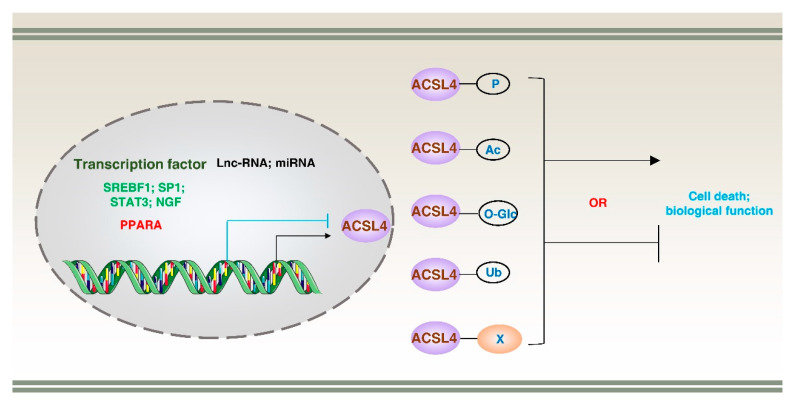
Transcriptional regulation of ACSL4. ACSL4 expression can be regulated by multiple transcription factors and non-coding RNA molecules (such as lncRNA and miRNA). Additionally, ACSL4 can undergo various post-translational modifications, including ubiquitination (Ub), phosphorylation (P), acetylation (ac), and O-GlcNAcylation (O-Glc), as well as engage in protein–protein interactions. These regulatory mechanisms play a crucial role in modulating cell death and various biological functions.

The activity and function of ACSL4 are regulated at both the transcriptional and post-translational modification (PTM) levels under various physiological and pathological conditions. These modifications can regulate the stability, localization, and interaction partners of ACSL4, thereby altering its catalytic activity and biological function. In the following sections, we will describe ACSL4’s transcriptional regulation, PTMs, and binding partners (Figure 4).

### 5.1. Transcriptional Regulation

The transcriptional regulation of the *ACSL4* gene is complex and involves multiple levels of regulation, including transcription factors [56], long non-coding RNA (lncRNA) [57], and microRNA (miRNA) [58]. Various internal and external factors can bind the promoter region of the *ACSL4* gene and promote or repress its transcription. For instance, nerve growth factor (NGF) [53], sterol regulatory element binding transcription factor 1 (SREBF1) [52], signal transducer and activator of transcription 3 (STAT3), and SP1 promote the transcription of *ACSL4* gene [36,59]. On the other hand, peroxisome proliferator activated receptor alpha (PPARA) represses the transcription of the *ACSL4* gene. PPARA downregulation reduces fatty acid binding protein 1 (FABP1) expression, promoting ACSL4 levels, which causes ferroptosis in human mesangial cells (HMCs) [31]. Additionally, non-coding RNAs, such as lncRNA and miRNA, can suppress or promote the expression of ACSL4. For example, lncRNA H19 protects against intracerebral hemorrhage injuries by the mi-106b-5p–ACSL4 axis [57]. Similarly, miR-20a-5p inhibits ACSL4-dependent ferroptosis by targeting the 3’ untranslated region of *ACSL4* mRNA, thereby preventing ischemia–reperfusion injury after renal transplantation [60]. Further understanding of the upstream signaling pathways that activate or repress ACSL4 transcription in response to various cellular and environmental cues is essential for targeting ACSL4 at the transcriptional level.

### 5.2. Post-Translational Modifications

#### 5.2.1. Phosphorylation

Protein phosphorylation is a PTM process in which a phosphate group is covalently added to a specific amino acid residue (serine, threonine, or tyrosine) in a protein. Several phosphorylation sites have been identified, and their importance in the regulation of ACSL4 is summarized in Table 2. Phosphorylation impacts the subcellular distribution of ACSL4, thereby regulating its intracellular localization. This modification is mediated by the coordinated action of kinases and phosphatases, such as protein kinase AMP-activated catalytic subunit (PRKA; also known as AMPK), protein kinase C beta II (PRKCβII), and protein kinase C (PRKC) [61,62]. These kinases can directly or indirectly phosphorylate ACSL4 and modulate its subcellular localization or receptor recognition, consequently enhancing or inhibiting its catalytic activity. For example, lactate-rich hepatocellular carcinoma cells (HCCs) exhibit resistance to ferroptosis by promoting ATP production and inactivating AMPK, which leads to the upregulation of SREBF1 and stearoyl-CoA desaturase (SCD), thus enhancing monounsaturated fatty-acid production and ferroptosis resistance. In contrast, inhibiting solute carrier family 16 member 1 (SLC16A1) to block lactate uptake activates AMPK, which leads to the downregulation of SCD1 and promotion of ferroptosis. This mechanism may synergize with ACSL4 to enhance ferroptotic sensitivity. However, the specific pathway through which the phosphorylation level of ACSL4 is regulated remains unclear [63]. Similarly, PRKCβII senses initial lipid peroxidation and amplifies the lipid peroxidation associated with ferroptosis by phosphorylating and activating ACSL4 at Thr328 [62]. In contrast, cellular stress can activate PRKC, which inhibits the phosphorylation of ACSL4, thereby decreasing its activity and reducing fatty-acid synthesis [61]. Further studies are needed to determine whether mutations in ACSL4 phosphorylation sites affect lipid metabolism in vivo.

#### 5.2.2. Methylation

Methylation is an important PTM that can regulate the transcriptional level, translation, and stability of ACSL4. This epigenetic modification involves the addition of methyl groups to DNA or proteins, altering their structure and function. The key enzymes involved in methylation processes are DNA methyltransferase (DNMT) and the ten-eleven translocation (TET) enzyme, which plays a role in DNA demethylation. DNMT methylates the CpG sites on DNA, thereby inhibiting the expression of ACSL4. On the other hand, TET enzymes remove methyl groups, leading to increased expression of ACSL4 [67]. In tumor cells, the methylation pathway of ACSL4 is generally inhibited, leading to PTM changes or overexpression that promotes tumor cell proliferation. However, the methylation-dependent reduction in ACSL4 levels can also prevent oxidative damage in cardiac myocytes and inhibit cardiomyocyte ferroptosis [68]. Methylation-dependent inhibition of ACSL4 also has a dual role. For example, in the context of hypoxia, lncRNA-CBSLR is induced and acts as a protective factor for gastric cancer cells against ferroptosis. It accomplishes this by interacting with YTHDF2, an RNA-binding protein that recognizes and binds N6-methyladenine (m6A)-modified RNA molecules, thereby regulating RNA degradation, translation, and splicing. The interaction between lncRNA-CBSLR and YTHDF2 reduces the stability of CBS mRNA, which, in turn, leads to decreased ACSL4 methylation, protein polyubiquitination, and subsequent degradation [69]. Although this provides new insights into the regulatory mechanism of ACSL4, the specific methylation enzymes and sites for ACSL4 require further investigation.

#### 5.2.3. Acetylation

ACSL4 can undergo acetylation, a PTM that is mainly catalyzed by histone acetyltransferase (HAT) and histone deacetylase (HDAC) [70]. HAT adds acetyl groups to lysine residues of ACSL4, thereby increasing its activity and stability, while HDAC removes acetyl groups and reduces its activity and stability. Multiple signaling pathways regulate the acetylation state of ACSL4. For example, hexokinase 2 (HK2) enhances acetyl-CoA accumulation, promotes H3K27-acetylation modification of the ACSL4 promoter and enhancer, and induces ACSL4-dependent fatty-acid β-oxidation, enhancing maintenance and self-renewal of HCC stem cells [71]. Moreover, acetylation of key transcription factors can also regulate the expression of ACSL4. For instance, fatty-acid β-oxidation enhances STAT3 acetylation, upregulating ACSL4-dependent phospholipid biosynthesis, which in turn enhances mitochondrial membrane potential and protects cancer cells from apoptosis induction [59]. Metabolic abnormalities are a common cause of many human diseases, and acetylation modifications tend to occur in enzymes involved in metabolic pathways [72]. Further exploration of the acetylation function of ACSL4 could provide a potential avenue for tumor treatment and also offer an important foundation for drug development in other metabolic disorders.

#### 5.2.4. O-GlcNAcylation

O-GlcNAcylation is a type of PTM that involves the addition of a single N-acetylglucosamine (GlcNAc) sugar molecule to the hydroxyl group of serine or threonine residues on proteins. It is a reversible modification that is dynamically regulated by two enzymes: O-GlcNAc transferase (OGT), which adds the GlcNAc sugar, and O-GlcNAcase (OGA), which removes it [73]. ACSL4 has high expression in HCC, promoting HCC survival and inhibition of apoptosis through O-GlcNAcylation, which is dependent on solute carrier family 2 member 1 (SLC2A1) [74]. However, the specific O-GlcNAcylation modification sites on ACSL4 are currently unknown. O-GlcNAcylation is predominantly carried out in the nucleus, whereas ACSL4 is mainly localized in the ER, reflecting the critical role of the linkage between organelles in regulating the biological function and activity of ACSL4. In addition to promoting cancer development, O-GlcNAcylation also regulates genomic stability and is associated with the maturation of autophagosome [75]. For instance, O-linked β-N-acetylglucosamine (O-GlcNAc) transferase (OGT) mediates O-GlcNAcylation of the SNARE protein synaptosome-associated protein 29 (SNAP29), which regulates autophagy in a nutrient-dependent manner, expanding the trophic sensors of autophagy beyond the mechanistic target of rapamycin kinase (mTOR) and phosphatidylinositol 3-kinase catalytic subunit type 3 complexes (PIK3C3; also known as VPS34) [76]. ACSL4 is predominantly localized to the endoplasmic reticulum, which serves as a major source of biomembranes essential for autophagy initiation. Consequently, O-GlcNAcylation of ACSL4 may synergistically contribute to the coordinated functioning of organelles implicated in autophagosome formation.

#### 5.2.5. Ubiquitination

Ubiquitination is a process that regulates the degradation, localization, activity, and interaction of target proteins by binding them to ubiquitin in various biological processes [77]. The ubiquitination of ACSL4 is mainly catalyzed by ubiquitin-activating enzyme (E1), ubiquitin-conjugating enzyme (E2), ubiquitin ligase (E3), and deubiquitinating enzyme (DUB). Ubiquitination-dependent degradation of ACSL4 has a dual role. For example, membrane-associated ring-CH-type finger 6 (MARCHF6), an E3 ubiquitin ligase, interacts with nicotinamide adenine dinucleotide phosphate (NADPH) to enhance its own activity while mediating the degradation of ACSL4 to inhibit ferroptosis in tumor cells [78]. However, ACSL4 is critical for arachidonic acid metabolism in tumor cells. ACSL4 is negatively correlated with the prognosis of HCC patients because it can stabilize the oncoprotein MYC proto-oncogene, BHLH transcription factor (MYC), in a mitogen-activated protein kinase (MAPK)/F-box and WD repeat domain containing 7 (FBXW7)-dependent manner via the ubiquitin-proteasome system, thus promoting cancer cell growth [79]. In addition, the ubiquitin-mediated degradation of ACSL4 protects some non-reproducible cells from ferroptosis. For example, prokineticin 2 (PROK2) accelerates F-box protein 10 (FBXO10)-driven ubiquitination, degrades ACSL4, and inhibits the biosynthesis of the lipid peroxidation substrates, arachidonic acid-phospholipids, thereby preventing neuronal cell death [80]. The upregulation of USO1 vesicle transport factor (USO1; also known as p115), induced by arachidonic acid or a high-fat diet, enhances its binding to ACSL4 and promotes its degradation [81]. Several ubiquitination sites of ACSL4 have been identified, including Lys113, Lys388, Lys397, Lys401, Lys500, Lys621, Lys670, and Lys702. However, the precise mechanism by which the ubiquitin proteasome system regulates ACSL4 through these sites, thereby impacting its biological function, remains unclear.

Regardless, the wide range of ubiquitin modification sites in ACSL4 offers potential for targeting in a similar manner to proteolysis targeting chimera (PROTAC) technology. However, the current focus of PROTAC research is on cytosolic proteins, and no significant breakthroughs have been achieved for membrane and organelle proteins [82]. Given that ACSL4 is primarily distributed in mitochondria and the ER, further exploration of ubiquitin degradation pathways and different ubiquitin modification sites in ACSL4 could accelerate clinical translation.

### 5.3. Protein–Protein Interaction

ACSL4–protein interactions are regulated by a complex process involving multiple molecular mechanisms that mediate different biological functions. Under normal conditions, the synthesis and degradation of ACSL4 maintain an equilibrium to ensure the orderly performance of cellular activities. When the balance between the two is disturbed, cell death can be induced. For example, ACSL4, a substrate for chaperone-mediated autophagy (CMA), binds to the receptor heat shock protein family A (Hsp70) member 8 (HSPA8) and translocates it to lysosomes for degradation. However, high glucose-induced glial maturation factor-β (GMFB) alkalizes lysosomes and disrupts autophagy, leading to the abnormal accumulation of ACSL4 and inducing ferroptosis in retinal pigment epithelium (RPE) cells [83]. In addition to lysosome-dependent autophagic degradation, proteasome-dependent ACSL4 degradation is another important pathway that maintains its dynamic equilibrium. A key step in this pathway is the interaction of ubiquitin ligases with ACSL4, as discussed in the section on “Ubiquitination”.

Another fruitful area of research is the study of ACSL4-mediated ferroptosis, which is affected by its binding proteins. For example, the gut microbiota metabolite glycochenodeoxycholate promotes transferrin receptor (TFRC) binding to ACSL4 and induces ferroptosis-dependent disruption of lipid metabolism and inflammation [84]. Similarly, TNF alpha-induced protein 3 (TNFAIP3; also known as A20)-binding to ACSL4 promotes ferroptosis in lung cancer A549 cells [85]. Additionally, some proteins are predicted or implied to interact with ACSL4, such as ATPase H^+^ transporting V0 subunit D1 (ATP6V0D1) [86], ACSL3 [87], KRAS proto-oncogene, GTPase (KRAS) [88], stimulator of interferon response cGAMP interactor 1 (STING1) [89], and forkhead box A3 (FOXA3) [90]. However, the impact of these binding partners on ACSL4 function is still poorly understood.

## 6. ACSL4 in Apoptosis

Apoptosis is crucial for the normal development and maintenance of tissue homeostasis. There are two main pathways that can activate apoptosis: the extrinsic pathway and the intrinsic pathway. The extrinsic pathway is activated by the binding of extracellular death ligands, such as tumor necrosis factor (TNF) and Fas ligand (FASLG), to their corresponding receptors on the cell surface. This leads to the formation of a death-inducing signaling complex (DISC), which recruits and activates caspase-8, a key initiator caspase that cleaves and activates downstream executioner caspases, such as caspase-3, leading to apoptosis [91]. The intrinsic pathway, also known as the mitochondrial pathway, is activated by various intracellular stress signals, such as DNA damage, oxidative stress, and growth factor deprivation. These signals trigger the release of cytochrome c from the mitochondria into the cytosol, where it forms a complex with apoptotic peptidase-activating factor 1 (APAF1) and caspase-9, forming the apoptosome complex. This complex then activates caspase-9, which cleaves and activates downstream executioner caspases, leading to apoptosis [92].

**Figure 5:**
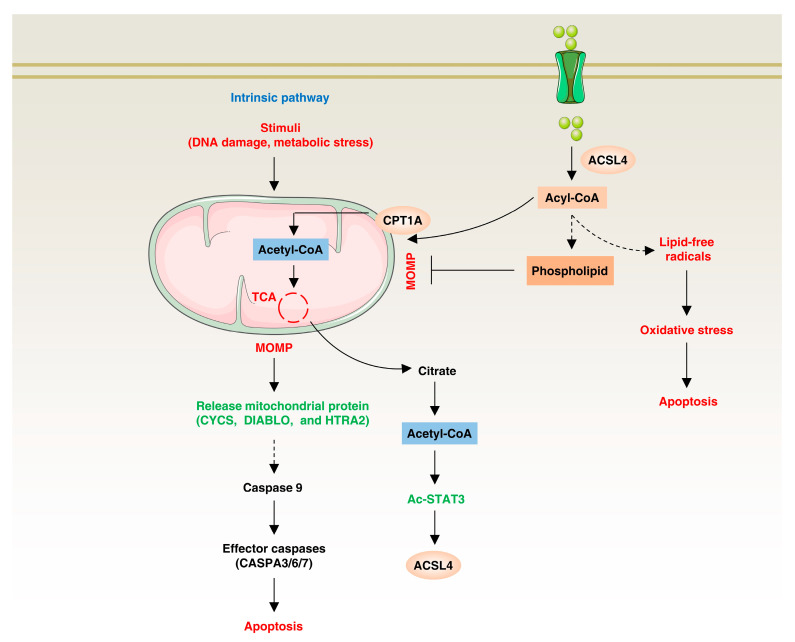
ACSL4 in apoptosis. On one hand, ACSL4 promotes apoptosis resistance by synthesizing lipids to increase mitochondrial membrane stability and by upregulating fatty-acid β-oxidation through ACSL4 expression. On the other hand, ACSL4 can also catalyze the synthesis of fatty acyl-CoA, which can generate lipid-free radicals and cause oxidative stress.

The mechanism by which ACSL4 is involved in regulating apoptosis is mainly by regulating the synthesis and metabolism of lipid metabolites, thereby affecting cell survival and death (Figure 5) ACSL4 is highly expressed in some cells and can inhibit apoptosis. For example, fatty-acid oxidation triggers the activation of STAT3 through the acetylation of coenzyme A (CoA), and the acetylated STAT3, in turn, upregulates the expression of ACSL4. This upregulation leads to increased phospholipid synthesis, resulting in elevated levels of mitochondrial membrane phospholipids. This, in turn, promotes enhanced mitochondrial integrity, enabling tumor cells to overcome chemotherapy-induced apoptosis [59]. In contrast, ACSL4 can catalyze the synthesis of fatty acyl-CoA to produce lipid free radicals, which can cause oxidative stress in cells, destroy important structures such as cell membrane, protein and DNA, and promote apoptosis [93,94]. For example, in the context of focal cerebral ischemia, ACSL4 is upregulated in a microRNA (miR-347)-dependent manner, consequently promoting neuronal apoptosis [95]. Additionally, ACSL4 directly interacts with and regulates proteins involved in apoptotic signaling pathways [96]. Thus, ACSL4 has a dual role in apoptosis: it can inhibit apoptosis by promoting fatty-acid oxidation, leading to increased phospholipid synthesis and chemoresistance, and it can promote apoptosis by producing lipid-free radicals and directly regulating apoptotic signaling pathways.

## 7. ACSL4 in Ferroptosis

Ferroptosis is a type of regulated necrosis that is characterized by the iron-dependent accumulation of lipid peroxides, resulting in damage to the plasma membrane and other cellular structures. Ferroptosis is mainly triggered by the depletion of glutathione peroxidase 4 (GPX4) activity, which leads to an accumulation of lipid peroxides and reactive oxygen species (ROS) in the cell. The process of ferroptosis is regulated by a complex network of pathways, including the metabolism of lipids, iron, and amino acids, as well as the activity of various enzymes and signaling pathways. Impaired ferroptosis is implicated in various physiological and pathological processes, including cancer, neurodegeneration, and ischemic tissue injury [97].

**Figure 6:**
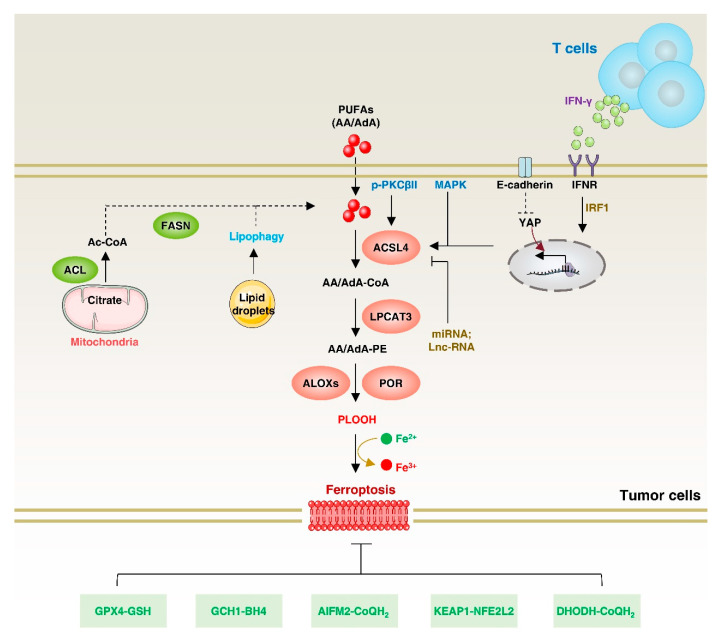
ACSL4 in ferroptosis. Exogenous and endogenous fatty acids undergo lipid peroxidation through ACSL4 to promote ferroptosis. The expression of ACSL4 is regulated by various pathways, including non-coding RNA, PKCβII, MAPK, and E-cadherin. Additionally, IFNγ and arachidonic acid from T cells in the tumor microenvironment promote ACSL4-dependent ferroptosis. On the other hand, there are multiple antioxidant pathways to inhibit ferroptosis. ACL: ATP-citrate lyase; POR, cytochrome p450 oxidoreductase; FASN, fatty acid synthase; GSH, glutathione; GCH1, GTP cyclohydrolase 1; BH4, tetrahydrobiopterin; AIFM2, apoptosis-inducing factor mitochondrial 2; CoQH_2_, ubiquinol; KEAP1, kelch-like ECH-associated protein 1; NFE2L2, nuclear factor erythroid 2-related factor 2; DHODH, dihydroorotate dehydrogenase.

At the molecular level, ferroptosis is characterized by the interplay between lipid peroxidation and the antioxidant system. PUFAs have a chemically unstable diallyl fraction that makes phospholipids containing PUFAs particularly vulnerable to peroxidation by redox-active iron. However, PUFAs cannot be directly incorporated into phospholipids; they must first be linked to CoA via acyl-CoA synthase to generate PUFA–CoA esters, which are then esterified to glycerol-3-phosphate to produce PUFA-containing phospholipids [98]. ACSL4 preferentially utilizes PUFAs such as arachidonic acid (AA) and eicosapentaenoate as substrates [99,100]. Functionally, ACSL4 performs different roles in regulating ferroptosis, making it a potential target for cancer therapy (Figure 6). For example, ACSL4 plays a role in facilitating PUFA-mediated plasma membrane lipid peroxidation, particularly in response to GPX4 and SLC7A11 inhibition, thereby promoting ferroptosis [101]. Consequently, inhibiting ACSL4 can potentially prevent ferroptosis under specific conditions.

However, ACSL4-dependent ferroptosis can also play a protective role in certain contexts for cancer cells. For example, during chronic injury-dependent HCC formation, ACSL4 deletion effectively slowed hepatocyte cell death and proliferation, inhibiting HCC formation by attenuating liver injury and hepatic fibrosis [102]. Similarly, myeloid-derived immunosuppressive cells (PMN-MDSCs) in the tumor microenvironment can spontaneously undergo ferroptosis, inducing the release of arachidonic-acid-oxidized lipids that inhibit T-cell activity [103].

While ACSL4 is considered a universal regulator of ferroptosis, it is not essential in SLC7A11- or cysteine-starvation-induced ferroptosis compared with GPX4 inhibition-dependent ferroptosis [104,105]. This difference may involve different types of PUFA-containing phospholipids and could be further explored through lipidomic studies. Additionally, the mechanisms by which ACSL4 mediates the propagation of plasma membrane lipid peroxidation in RSL3-dependent ferroptosis are still not fully understood. Further investigation into ACSL4-mediated pathways could provide new insights into the prevention and treatment of related diseases.

## 8. ACSL4 in Autophagy

**Figure 7:**
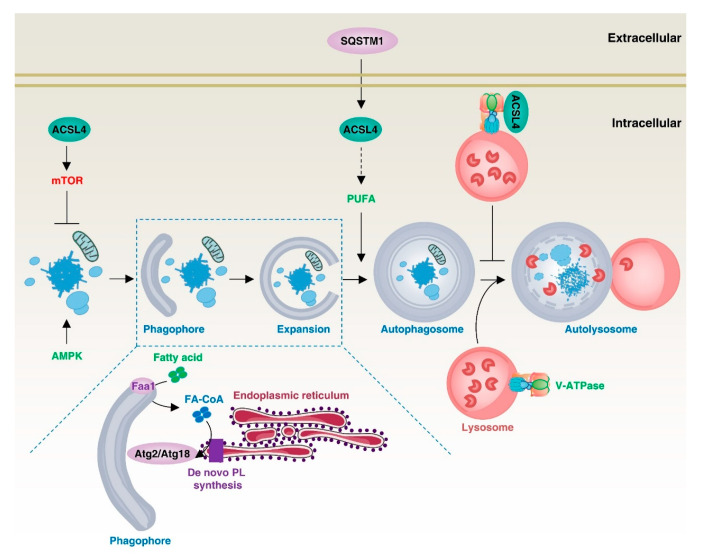
ACSL4 in autophagy. ACSL4 can regulate the autophagy process through different mechanisms. It can inhibit autophagy by promoting mTOR expression or binding to V-ATPase. On the other hand, Faa1, located in the phagophore, promotes de novo synthesis of fatty acids, and extracellular SQSTM1-dependent ACSL4 expression promotes autophagy.

Autophagy is a lysosome-mediated intracellular degradation mechanism that removes unwanted organelles, proteins, or other macromolecules from cells and recycles them. At the molecular level, autophagy relies on a series of autophagy-related (ATG) proteins to regulate the formation of multiple membrane structures, including phagophores, autophagosomes, and autolysosomes [106]. In the process of autophagy, lipid molecules produced by lipid metabolism can act as components of the autophagosome and participate in the autophagic process (Figure 7).

In yeast, the acyl-coenzyme A synthase aortic aneurysm, familial thoracic 1 (AAT1; also known as FAA1) accumulates in nucleated autophagic vesicles and locally activates the fatty acids required for autophagy vesicle elongation and autophagy, promoting the assembly of autophagic membranes [13]. In addition, yeast FAA4 mediates the symbiosis between lipid droplets and the ER and promotes the expansion of lipid droplets. Furthermore, when lipolysis is induced by refreshed medium, FAA4 shuttles to vacuoles through the ER and lipophagy, where it may activate FAs for membrane expansion and degrade FAA4. However, there is currently insufficient valid evidence regarding the degradation mechanism of FAA4 in the vacuole [107]. The acyl-CoA synthetase ACSL4 protein in mammalian cells has also been shown to be localized to nascent autophagosomes and to regulate the homeostasis of autophagic flux [13].

ACSL4 can also regulate intracellular autophagic signaling pathways, such as the AMPK and mTOR pathways, and thus promote or inhibit autophagy. For example, angiotensin II (Ang II) promotes the activation of the mitochondrial mTOR1/2 signaling protein and promotes the proliferation of human H295R adrenocortical cells in an ACSL4-dependent manner [108]. Similarly, ACSL4 promotes ferroptosis in SH-SY5Y cells through the AMPK/mTOR pathway, although the detailed mechanism remains unclear [109]. The formation of autophagosomes and lysosomes requires an acidic lysosomal environment, which depends on V-ATPase activity. ACSL4 can bind to V-ATPases to inhibit their activity, thereby inhibiting the formation of autophagosomes [85,110]. Autophagy levels also regulate ACSL4 and therefore affect the sensitivity to ferroptosis. For example, the autophagy receptor protein sequestosome 1 (SQSTM1) increases advanced glycosylation end-product-specific receptor (AGER)-dependent ACSL4 expression, leading to PUFA production for autophagosome formation and subsequent ferroptosis, which promotes acute pancreatitis [111]. These findings provide an important reference for an in-depth understanding of the relationship between intracellular lipid metabolism, autophagy, and inflammation.

## 9. ACSL4 in Diseases

**Figure 8:**
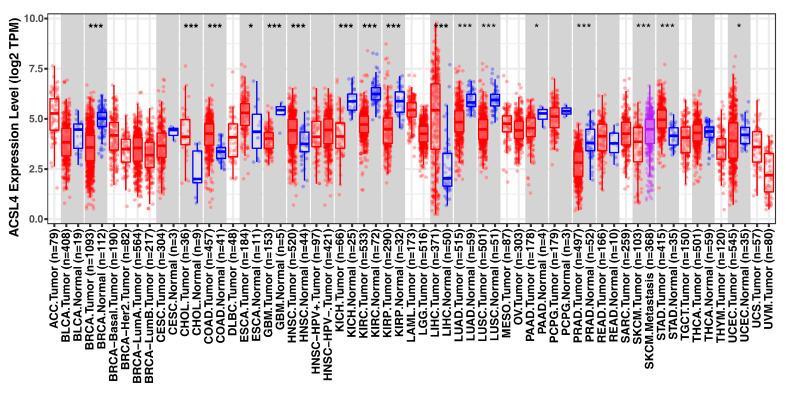
The expression of ACSL4 in different tumors. *: *p* < 0.05; ***: *p* < 0.001. Data from http://timer.cistrome.org/ (accessed on 29 March 2023).

Alterations in ACSL4′s activity and expression contribute to the development and progression of various diseases, such as obesity, acute kidney injury (AKI), neurodegenerative diseases, cardiovascular diseases, exertional heat stroke, non-alcoholic fatty liver disease (NAFLD), and cancer (Table 3; Figure 8). In the following sections, we will discuss how ACSL4 is involved in the development of these diseases.

### 9.1. Obesity

Obesity is a prevalent metabolic syndrome, regulated by both external and internal factors. The main external factor is excessive caloric intake resulting in increased fat synthesis, while internal factors are mainly disorders of fat metabolism [136,137]. ACSL4 controls the occurrence and development of obesity mainly by regulating fatty-acid metabolism, mitochondrial function, and hormone secretion. For instance, adipocyte-specific knockout of *Acsl4* in mice (Ad-KO mice) protects against high-fat diet-induced obesity, inhibits adipocyte death, and effectively prevents increased fat and liver fat accumulation by inducing gonadal white adipose tissue oxygen consumption and whole-body energy expenditure [112]. In early pregnancy, obesity leads to ovarian dysfunction, which is closely associated with ACSL4-mediated fatty-acid synthesis [113]. ACSL4 also plays a role in steroid hormone synthesis, as evidenced by the reduced production of corticosterone and testosterone in steroid-tissue-specific *Acsl4* knockout mice, resulting in altered cholesteryl ester composition [115].

Furthermore, in both in vivo mouse models and human cardiomyocytes deficient in the mitochondrial autophagy receptor FUN14 domain-containing 1 (FUNDC1), a short-term high-fat diet (HFD) exacerbates cardiac remodeling and contraction dysfunction through ACSL4-mediated regulation of ferroptosis [114]. These findings suggest that FUNDC1- and ACSL4-dependent ferroptosis may serve as a target for early obesity-induced cardiac dysfunction. In addition, a transcriptomic study of flaxseed polysaccharide (FP)-treated rats also suggests an important role for ACSL4 in the development of obesity [138].

In summary, ACSL4’s association with obesity is mainly manifested in its effect on fatty-acid metabolism. In addition to limiting excessive calorie intake from exogenous sources, future modification of ACSL4 through gene-editing technology without affecting its normal function may be an effective way to treat obesity.

### 9.2. Ischemia–Reperfusion Disease

Ischemia–reperfusion disease is a condition that occurs when blood flow to a particular part of the body is reduced or completely stopped for a period of time, followed by the restoration of blood flow. This can lead to damage and dysfunction in various organs, such as the heart, brain, and systemic tissues, arising from the deprivation of oxygen and nutrients during the ischemic phase, as well as the subsequent inflammation and oxidative stress during the reperfusion phase [139]. ACSL4 overexpression can lead to the synthesis and metabolism of fatty acids, which may contribute to the development of ischemia–reperfusion disease. Mechanistically, this also involves the regulation of abnormal oxidative stress, the inflammatory response, and cell death pathways.

For instance, intestinal ischemia triggers ACSL4-dependent ferroptosis-mediated tissue injury during reperfusion. However, inhibiting ACSL4 in human Caco-2 cells using genetic approaches (such as siRNA) or in vivo ACSL4 inhibition with rosiglitazone (ROSI) in mice effectively mitigates the effects of ischemia and hypoxia. ROSI-fed mice are protected against ischemia–reperfusion injury, with a mitigating effect on intestinal barrier dysfunction, and they exhibited restored GPX4 expression, which reduced mitochondrially encoded cytochrome C oxidase II (MT-CO2) expression and lactate dehydrogenase (LDH) levels [33]. Multiple regulatory factors can induce or aggravate ischemia–reperfusion injury through the ACSL4 pathway. Thrombin stimulates ferroptosis signaling by promoting the mobilization and subsequent esterification of arachidonic acid via ACSL4 during cerebral ischemia/reperfusion [121]. LncAABR07025387.1 enhances myocardial ischemia–reperfusion injury through the miR-205/ACSL4 axis [118]. The inhibition of ACSL4 effectively ameliorates ischemia–reperfusion injury through different drugs or non-coding RNAs [60,117,123,140].

Similarly, ACSL4 has been shown to be a key regulator of stroke, with inhibition of ACSL4 in vivo improving brain injury and neuroinflammation [141]. In contrast, overexpression of ACSL4 aggravates ischemic brain damage [142]. Lipid metabolism disorder is the basis of atherosclerosis, and although the link between ferroptosis and atherosclerosis is not fully understood, there is evidence of significant upregulation of ACSL4 protein in tissue samples from atherosclerosis patients [143,144]. Furthermore, rosiglitazone inhibits ACSL activity and fatty-acid partitioning in human and mouse arterial smooth muscle cells and human macrophages, suggesting that ACSL4 may regulate fatty-acid partitioning and the biological effects of atherosclerosis [145]. In addition, ACSL4 mediates mitochondria-dependent ferroptosis and damages the cardiac microvasculature in diabetic patients, whereas pharmacological inhibition or overexpression of the ACSL4 negative regulator mitofusin 2 (MFN2) by isorhapontigenin attenuates mitochondrial dysfunction and protects the diabetic cardiac microvasculature [146].

### 9.3. Nonalcoholic Fatty Liver Disease (NAFLD)

NAFLD is a condition where there is an abnormal accumulation of fat in the liver that is not caused by excessive alcohol consumption. It is a spectrum of liver diseases that ranges from simple steatosis (fatty liver) to non-alcoholic steatohepatitis (NASH), which is a more severe form of the disease that can progress to liver fibrosis, cirrhosis, and even liver cancer. NAFLD is commonly associated with obesity, insulin resistance, and metabolic syndrome, and it is becoming increasingly prevalent worldwide [147]. Numerous studies have demonstrated that the expression of ACSL4 in liver tissue of patients with NAFLD is significantly increased. Liver-specific knockout of *acsl4* in mice ameliorates steatosis and liver fibrosis. Moreover, abemaciclib, a potent and selective inhibitor of ACSL4, alleviates most of the NAFLD symptoms in NAFLD mice. Mechanistically, inhibition of ACSL4 promotes mitochondrial respiration, thereby enhancing hepatocyte-mediated fatty-acid β-oxidation and reduces lipid accumulation through the upregulation of PPARA [128]. Similarly, rosiglitazone or siRNA-mediated inhibition of arsenic exposure-induced ACSL4 in rat liver and L-02 cells attenuates the ferroptotic response and NASH [148]. ACSL4 is also implicated in pathological processes such as the inflammatory response and liver fibrosis in the liver [149]. Nevertheless, the detailed mechanism of ACSL4 and NAFLD development remains elusive, and exploring the interaction between ACSL4 and different proteins in NAFLD development at multiple levels, including molecular, cellular, and animal models, is an important area of future research.

### 9.4. Neurodegeneration

Neurodegenerative diseases refer to a group of neurological disorders, including Alzheimer’s disease, Parkinson’s disease, Huntington’s disease, and multiple sclerosis. These diseases share the common feature of neuronal death and loss of neurological function [150]. ACSL4 expression is elevated in patients with these diseases, and overexpression of ACSL4 leads to neuronal death and loss of neurological function. The link between ACSL4 and neurodegenerative lesions may be related to its role in lipid metabolism. ACSL4 catalyzes the binding of long-chain fatty acids to CoA to form fatty acyl-CoA, a metabolite that may cause oxidative stress and cell death in neurons [151]. Additionally, ACSL4 is involved in the construction and maintenance of neuronal membranes, and its overexpression may lead to the instability and dysfunction of neuronal membranes [142].

ACSL4 regulates neurodegeneration mainly through inflammation and cell death pathways. For example, increased ACSL4 expression induced ferroptosis in experimental autoimmune encephalitis (EAE) mice by promoting T-cell-mediated cytotoxicity and inflammation. Targeting ferroptosis with the ferroptosis inhibitor ceruloplasmin or inhibiting ACSL4 expression improved the behavioral phenotype of EAE mice, attenuated neuroinflammation, and prevented neuronal death [133]. Similarly, lipopolysaccharide (LPS)-induced ACSL4 promotes neuroinflammation-dependent Parkinson’s disease (PD). The knockdown of *Acsl4* in the microglia reduces the production of pro-inflammatory cytokines in vitro and attenuates LPS- and acute MPTP-induced neuroinflammation in vivo. Mechanistically, ACSL4 promotes NF-κB signaling by decreasing the expression of residue-like family member 4 (VGLL4) [35].

Some drugs and regulatory factors have rescued neurodegenerative diseases by inhibiting ACSL4-dependent ferroptosis. For example, tetrahydroxy stilbene glycoside dose-dependently reduces resistance to amyloid beta peptide (Aβ)-induced neuronal cytotoxic death by decreasing ferroptosis-associated proteins, including ACSL4 [152]. In addition, inhibition of ACSL4 similarly ameliorates secondary disease induced by neurodegenerative lesions. For example, aldehyde dehydrogenase 2 family member (ALDH2) ameliorated cardiac systolic dysfunction in an amyloid precursor protein (APP)/presenilin 1 (PS1) murine model of Alzheimer’s disease by inhibiting ACSL4-dependent ferroptosis [130]. These findings suggest that ACSL4 is a potential therapeutic target in neurodegeneration. However, a significant challenge is the development of small molecules that can cross the blood–brain barrier.

### 9.5. Exertional Heat Stroke

Exertional heat stroke (EHS) is an extreme exercise-related disease caused by high body temperature resulting from strenuous exercise in a high-temperature environment. EHS can lead to an imbalance in body-heat balance and a series of severe physiological reactions, and rhabdomyolysis (RM) is one of the common complications [153]. Studies have shown that the expression level of ACSL4 is significantly elevated in EHS patients, leading to alterations in the lipid composition of cell membranes, which increases the permeability and fragility of cell membranes, inducing cell-membrane rupture and cell death. For example, EHS induces ACSL4 expression and promotes ferroptosis-dependent RM, whereas the development of RM in mice was significantly inhibited by the use of ferroptosis inhibitors (such as ferrostatin-1) after EHS. In vitro experiments have also shown that inhibition of ACSL4 with rosiglitazone significantly inhibits lipid peroxidation and its metabolites. Mechanistically, ACSL4-mediated RM is Yes-associated protein (YAP)-dependent via TEA domain transcription factor 1/TEA domain transcription factor 4 [134].

Heat-shock proteins are important regulators of the intracellular stress response, especially to heat stress. Some key heat-shock proteins, such as heat-shock protein family A (Hsp70) member 5 (HSPA5), have been shown to be involved in the regulation of ferroptosis [154]. Further understanding of the role of ACSL4 in heat stress and its regulatory mechanisms may provide new ideas for the prevention and treatment of EHS.

### 9.6. Acute Kidney Injury

ACSL4 is widely expressed in the kidney, especially in renal tubular epithelial cells. Acute kidney injury (AKI) is a disease in which kidney function suddenly decreases and is commonly caused by acute conditions such as renal ischemia, nephrotoxic drugs, and infections [155]. ACSL4 plays an important role in AKI, which is reflected at several levels. First, ACSL4 is involved in the lipid metabolism of renal tubular cells. In AKI, renal tubular cells are damaged, leading to the disruption of lipid metabolism, including the oxidative stress-dependent lipid peroxidation of cell membranes, and this induces cell death [126,156]. Second, ACSL4 is involved in the inflammatory response of renal tubular cells. Renal tubular cells are stimulated by inflammatory factors, leading to an increased intracellular inflammatory response, which causes cell death [157,158]. For example, the renal tubular *Acsl4* knockout, as in Cdh16^Cre^-ACSL4^F/F^ mice, had significantly reduced ferroptosis and inhibited functional and pathological impairment in AKI mice. Meanwhile, renal inflammation and macrophage infiltration are significantly reduced in Cdh16Cre-ACSL4^F/F^ mice. Mechanistically, the downregulation of hypoxia-inducible factor 1 subunit alpha (HIF1A) promotes the upregulation of ACSL4 in AKI [126]. XJB-5-131, identified as a promising protective agent against renal tubular injury, attenuated ischemia–reperfusion-induced renal injury and inflammation in mice by specifically inhibiting ACSL4-dependent ferroptosis but not necroptosis and pyroptosis [123]. In contrast, a study showed that ferroptosis and necroptosis synergistically manipulate cell death in acute injury. In particular, ACSL4 is initially upregulated during AKI, and its expression level correlates with the severity of tissue injury, suggesting that inhibition of ACSL4-dependent ferroptosis can control the progression of disease in the early stages of AKI [159].

### 9.7. Cancer

Cancer is a multifaceted disease that involves various factors in its development and progression, including genetic mutations, imbalanced cell proliferation and death, and angiogenesis [160]. ACSL4 is significantly upregulated in tumor cells, particularly on the membranes of tumor cells. Overexpression of ACSL4 is believed to promote tumor cell proliferation and invasion (Figure 8) [161,162]. The association between ACSL4 and cancer is mainly demonstrated at several levels: (1) promotion of lipid synthesis required for tumor cell growth, (2) regulation of apoptosis signaling pathways and cell cycle protein expression, and (3) regulation of the tumor immune microenvironment to mediate immune evasion. 

However, on the other hand, ACSL4 is a potential anti-cancer target. Induction of ACSL4-dependent ferroptosis is a promising therapeutic option for several cancers [163]. For example, targeting ACSL4 inhibits colorectal cancer (CRC) proliferation and prolongs survival time in CRC mice [161]. Similarly, ACSL4 promotes HCC growth and enhances cancer cell stemness through lipid metabolism. Mechanistically, ACSL4 upregulates SREBF1 and its downstream lipogenic enzymes in HCC cells via Myc [52]. Conversely, targeting ACSL4-dependent ferroptosis effectively kills cancer cells, especially apoptosis-evading cells. For example, interferon γ (IFNγ) from CD8^+^ T cells combined with arachidonic acid from the microenvironment induces immunogenic tumor ferroptosis. The cytotoxic T-cell (CTL)-mediated killing mode is dependent on IFNγ-stimulated ACSL4 expression, which in turn increases the binding of palmitoleic acid and oleic acid to phospholipids. This ultimately leads to CTL-induced ferroptosis in tumor cells [164].

Collectively, ACSL4 plays a dual role in tumorigenesis, participating in lipid metabolism, cell proliferation, and regulation of the tumor microenvironment on the one hand, and mediating cellular ferroptosis on the other hand. ACSL4-dependent ferroptosis has been considered an effective treatment for apoptosis-resistant cancers. However, cancer cells can also evade ferroptosis by utilizing metabolic reprogramming, such as the pyruvate dehydrogenase kinase 4 (PDK4)-mediated inhibition of pyruvate oxidation and fatty-acid synthesis [165]. Therefore, understanding the ACSL4-mediated switch between pro-death and anti-death mechanisms will be a crucial step towards developing more effective cancer therapies.

## 10. ACSL4-targeted Drugs

The development and application of these compounds provide new ideas and strategies for studying the biological functions of ACSL4 and treating related diseases. Here, we will discuss some key inducers and inhibitors and their functions (Table 4).

### 10.1. Inducers

Some endogenous fatty acids or hormones have the ability to induce ACSL4 expression or enhance its activity. For instance, eicosapentaenoate ethyl, a component of fatty acids, acts as a precursor for the prostaglandin-3 and thromboxane-3 families, leading to lower lipid concentrations and preventing platelet aggregation, ultimately reducing the risk of cardiovascular events in patients with hypertriglyceridemia [180]. A study has shown that eicosapentaenoic acid-induced apoptosis is dependent on ACSL4 [170]. On the other hand, 17-β-estradiol induces estrogen receptor 1 (ESR1)-positive breast cancer proliferation and invasive capacity through altered cellular lipid metabolism, resulting in cellular uptake of the polyunsaturated fatty acids AA and eicosapentaenoic acid (EPA) and increased ACSL4 protein levels through non-transcriptional means [173]. However, the exact mechanism through which EPA activates ACSL4 is yet to be fully understood. Moreover, some endogenous regulators can induce ACSL4 expression, thereby promoting the progression of related diseases. For instance, thrombin induces ACSL4-dependent ferroptosis during cerebral ischemia/reperfusion [121]. ACSL4 is also abundant in steroidogenic tissues, and its expression is induced by adrenocorticotropic hormone (ACTH) in a transcription-independent manner and repressed by glucocorticoids [181,182]. Furthermore, some inducers of ferroptosis can indirectly induce the expression of ACSL4 in specific settings, such as Erastin and RSL3, which may involve the participation of polyunsaturated fatty acids [6,98]. Given the dual role of ACSL4 in different pathologies, exploring inducers of ACSL4 could potentially provide therapeutic options for related diseases.

### 10.2. Inhibitors

In addition to genetic approaches, pharmacological inhibition of ACSL4 activity or expression is a critical intervention in the treatment of many diseases. These inhibitors can be classified into different classes based on their properties, including herbal products, cellular metabolites, negative regulatory factor agonists, and chemical synthesis products. For example, rosiglitazone can inhibit ACSL4 expression and activity by activating peroxisome proliferator activated receptor gamma (PPARG). In addition to improving glucose utilization and reducing blood glucose levels, rosiglitazone can also inhibit tumor cell proliferation and metastasis, sensitize tumor cells to chemotherapeutic drugs, or improve ferroptosis-dependent ischemia–reperfusion injury by regulating lipid metabolism [145,183]. Troglitazone, a PPAR agonist-dependent hypoglycemic agent, has been shown to suppress radiation-induced intestinal injury ferroptosis by inhibiting ACSL4 [176]. Triacsin C is a natural product and broad-spectrum ACSL inhibitor that inhibits ACSL4 activity and has potential therapeutic effects on various types of tumors and results in improvement in some neurodegenerative diseases [130,162,184]. Abemaciclib, a cyclin-dependent kinase 4 (CDK4) and CDK6 inhibitor, selectively inhibits ACSL4 and can significantly improve most NAFLD symptoms in a mouse model of NAFLD by promoting fatty-acid β-oxidation at low doses [128]. AA, as a substrate of ACSL4, can enhance the ubiquitination degradation of ACSL4, which can be blocked by specific inhibitors of the proteasome degradation pathway [175].

Moreover, since ACSL4 expression and activity are regulated by multiple regulators and different PTMs, derivatives of different inhibitors and inhibitors of these modification pathways can competitively inhibit ACSL4 expression. For example, valnoctamide, a non-teratogenic amide derivative of valproic acid, can non-competitively inhibit the activation of AA-CoA by ACSL4 and is suggested to be a therapeutic drug for bipolar disorders [178,179].

## 11. Conclusions and Future Perspectives

Lipid synthesis and metabolic pathways play a critical role in maintaining cellular life activity. ACSL4, a fatty-acid coenzyme A synthetase, is involved in various biological processes by regulating lipid metabolism. ACSL4 promotes lipid synthesis and regulates the composition of cellular membranes. It also plays a dual role in autophagy and ferroptosis, two cellular processes that have important implications in disease progression. Inhibition or induction of ACSL4 activity and expression through pharmacology or genetics can control or ameliorate disease progression. However, the multifunctional role of ACSL4 raises some questions that remain to be pursued in the future.

First, further exploration is needed to understand the mechanistic switch of ACSL4 in ferroptosis and its synergy with other regulatory cell death processes. Second, protein cryo-electron microscopy techniques are required to resolve the detailed structure of ACSL4 and its rapid transformation mechanism. Third, the detailed mechanism by which ACSL4 becomes a substrate predisposed to interact with other families requires further exploration. Fourth, more research is required to understand how ACSL4 is associated with other processes of autophagosome formation and how different regulatory factors affect the progression of autophagy. Fifth, exploring the self-amplifying effect of DAMPs released from ACSL4-dependent ferroptosis in the tumor microenvironment could provide new insights into tumor therapy. Finally, the development of tissue-localized specific inhibitors or inducers of ACSL4 is crucial for precision therapy as normal biological processes are also necessary for normal cells.

In summary, ACSL4 is a multifaceted protein with diverse functions in various biological processes, and it holds great potential as a therapeutic target for various diseases. Further exploration of the molecular mechanisms of ACSL4 regulation will deepen our understanding of its functions in different pathophysiological processes, providing a foundation for the future development of new drugs and therapies.

## Figures and Tables

**Table 1:** Characteristics of the ACSL family.

Name	Chromosome Localization	Size (Number of Amino Acids)	Molecular Mass (Da)	Preferential Substrate	Tissue Expression (Most Abundant)	Subcellular Distribution (Most Abundant)	Function
ACSL1	4q35.1	698	77,943	Palmitoleate, oleate and linoleate	Liver	Endoplasmic reticulum, mitochondrion	Catalyzes the conversion of long-chain fatty acids to their activated acyl-CoA forms for both cellular lipid synthesis and degradation via beta-oxidation.
ACSL3	2q36.1	720	80,420	Myristate, laurate, arachidonate and eicosapentaenoate	Nervous system	Golgi apparatus, endoplasmic reticulum	Activates long-chain fatty acids for both cellular lipid synthesis and degradation via beta-oxidation. Promotes hepatic lipogenesis and the incorporation of fatty acids into phosphatidylcholine.
ACSL4	Xq23	711	79,188	Arachidonate and eicosapentaenoate	Eye, stomach	Endoplasmic reticulum, mitochondrion, plasma membrane	Catalyzes the conversion of long-chain fatty acids to their activated acyl-CoA forms for both cellular lipid synthesis and degradation via beta-oxidation.
ACSL5	10q25.2	683	75,991	A wide range of saturated fatty acids and a preference for C16–C18 unsaturated fatty acids	Intestine	Endoplasmic reticulum, nucleus, mitochondrion, plasma membrane	Catalyzes the conversion of long-chain fatty acids to their activated acyl-CoA forms for both cellular lipid synthesis and degradation via beta-oxidation. Activates fatty acids from exogenous sources for the synthesis of triacylglycerol, which is destined for intracellular storage.
ACSL6	5q31	697	77,752	Equal preference for saturated and polyunsaturated fatty acids with a backbone of C16–C20	Nervous system	Endoplasmic reticulum, plasma membrane	Catalyzes the conversion of long-chain fatty acids to their activated acyl-CoA forms for both cellular lipid synthesis and degradation via beta-oxidation.

**Table 2:** Protein modification sites in ACSL4.

Type	Residue	Site	Ref
Phosphorylation	Ser	140	[64]
Phosphorylation	Ser	674	[65]
Phosphorylation	Ser	95	[64]
Phosphorylation	Thr	679	[65]
Acetylation	Lysine (K)	89	[66]

**Table 3:** ACSL4 in diseases.

Type	Expression	Phenotype and Mechanism	Ref.
Obesity	Upregulation	Promotes the participation of arachidonic acid in phospholipids, leading to hepatic fat accumulation, inflammation in gonadal white adipose tissue, and insulin resistance	[112,113]
Cardiac remodeling and contraction	Upregulation	Short-term high-fat diet intake leads to downregulation of FUNDC1 and upregulation of ACSL4, which can result in lipid peroxidation-dependent defects in cardiac geometry and function.	[114]
Steroidogenesis	Normal	Promotes the formation of adrenal cholesterol esters and determines the fatty acyl composition of these esters	[115]
Vascular disease	Upregulation	Promotes the synthesis and metabolism of arachidonic acid and inhibits the secretion of prostaglandin E_2_ in vascular cells	[116]
Intestinal ischemia/reperfusion	Upregulation	Ischemia induces the upregulation of the SP1–ACSL4 cascade, promoting ferroptosis-dependent intestinal reperfusion injury	[33]
Myocardial ischemia/reperfusion	Upregulation	Promotes myocardial ischemia/reperfusion injury through lipid peroxidation-dependent ferroptosis	[117,118]
Pulmonary ischemia/reperfusion	Upregulation	Promotes pulmonary ischemia/reperfusion injury through lipid peroxidation-dependent ferroptosis	[119]
Cerebral ischemia/reperfusion	Upregulation	Promotes cerebral ischemia/reperfusion injury through the GPX4–ACSL4–ACSL3 pathway	[120]
Ischemic stroke	Upregulation	Thrombin-induced activation of serine protease induces ACSL4-dependent ferroptosis in neuronal cells, leading to ischemic stroke	[121]
Renal ischemia/reperfusion injury	Upregulation	Promotes renal damage and inflammation related to ferroptosis	[122]
Acute kidney injury	Upregulation	Promotes ferroptosis in renal tubular epithelial cells, leading to inflammation and acute kidney injury	[123,124,125,126]
Acute lung injury	Downregulation	Isoliquiritin apioside inhibits HIF1A, leading to downregulation of ACSL4 and preventing acute lung injury caused by intestinal ischemia/reperfusion	[127]
Non-alcoholic fatty liver disease	Upregulation	Induces the development of hepatic steatosis and fibrosis	[128,129]
Alzheimer’s disease	Upregulation	Induces ferroptosis-dependent brain damage and increases cytoplasmic phospholipase A2 in the mouse cortex	[130,131]
Parkinson’s disease	Upregulation	Induces ferroptosis in the substantia nigra brain pathway and mediates the production of cytokines	[35,132]
Multiple sclerosis	Upregulation	Induces ferroptosis-dependent encephalitis	[133]
Exertional heat stroke	Upregulation	Promotes muscle cell death induced by exertional heat stroke via ferroptosis	[134]
X-linked intellectual developmental disorder	Mutation	Induces X-linked intellectual developmental disorder	[24,135]

**Table 4:** ACSL4-related reagents.

Type	Name	Mechanism	Ref
Inducer	Adrenocorticotropic hormone	Induces dephosphorylation of protein tyrosine phosphatase and the activity of ACOT2	[166,167,168]
Inducer	RSL3; Erastin	Inhibits the SLC7A11-GPX4 pathway	[6,169]
Inducer	Icosapent	Increases the level of endogenous polyunsaturated fatty acids	[170,171]
Inducer	Acyl-CoA	Induces dephosphorylation of protein tyrosine phosphatase and the activity of ACOT2	[168,172]
Inducer	17 β-estradiol	Promotes polyunsaturated fatty-acid uptake	[173]
Inducer	Thrombin	Promotes the mobilization of phosphatidylethanolamine (PE) and phosphatidylcholine (PC) in the neuronal cell membrane via cPLA2α	[121]
Inhibitor	Pinolenic acid	Increases the level of endogenous polyunsaturated fatty acids	[174]
Inhibitor	Rosiglitazone	PPARγ agonist	[33,145]
Inhibitor	Arachidonic acid	Promotes ACSL4 ubiquitination	[175]
Inhibitor	Troglitazone	PPARγ agonist	[176]
Inhibitor	Triacsin C	Broad-spectrum ACSL inhibitor	[162,177]
Inhibitor	Abemaciclib	ACSL4 inhibitor	[128]
Inhibitor	Valnoctamide	Derivative of valproate	[178,179]

## Data Availability

Not applicable.
